# Predictors of benefits and safety events from participation in a lifestyle therapy trial for adults with depression: A secondary analysis of the CALM randomised controlled trial

**DOI:** 10.1177/00048674261440324

**Published:** 2026-04-14

**Authors:** Lara K Radovic, Madeleine L Connolly, Lauren M Young, Marita Bryan, Wolfgang Marx, Megan Turner, Dean Saunders, Sophie Mahoney, Rachel Fiddes, Tayla John, Amelia McGuinness, Tetyana Rocks, Deborah N Ashtree, Adrienne O’Neil

**Affiliations:** 1 Food and Mood Centre, Institute for Mental and Physical Health and Clinical Translation (IMPACT), School of Medicine, Deakin University, Barwon Health, Geelong, VIC, Australia

**Keywords:** Lifestyle therapy, depression, behavioural intervention, predictors, safety events, treatment response

## Abstract

**Objective:**

Lifestyle-based interventions are increasingly popular for treating depression, yet a comprehensive evaluation of who benefits or may be harmed is limited. This study examined predictors of benefits and safety events, and the types of these events experienced by participants in the CALM trial, which compared lifestyle therapy with psychotherapy for depression.

**Methods:**

‘Benefit’ was defined as a ⩾ 50% reduction in Patient Health Questionnaire-9 scores, along with self-reported or staff-observed safety events. Generalised estimating equations identified predictors of benefit and safety events, reporting risk ratios and beta coefficients. Exploratory subgroup analyses were conducted by treatment arm (lifestyle vs psychotherapy).

**Results:**

Of 132 completers, 38% met criteria for benefit and 78% reported at least one safety event. Older age (RR = 1.14, 95% CI [1.01, 1.30]) and being born overseas (RR = 1.59, 95% CI [1.06, 2.38]) predicted benefit. Older age (β = 0.16, 95% CI [0.05, 0.26]) and higher baseline glucose (β = 0.16, 95% CI [0.10, 0.23]) were associated with more safety events. Subgroup analyses indicated that age predicted benefit in the psychotherapy arm, while place of birth predicted benefit in the lifestyle arm. Safety events were more common in the lifestyle arm among participants who were older or had elevated glucose.

**Conclusion:**

In the CALM trial, older age and being born overseas predicted benefit, while older age and higher glucose levels were associated with greater safety events. These findings provide clinicians and consumers with a clearer risk-benefit profile of behavioural therapies and support personalised treatment based on consumer characteristics.

**Registration:**

Australian and New Zealand Clinical Trials Registry (https://www.anzctr.org.au/; ACTRN12621000387820).

## Introduction

Common mental disorders persist as leading contributors to disease burden globally (World Health Organisation, 2022). Despite an increase in the use of conventional treatments, such as psycho- and pharmacotherapy (Correll et al., 2023; Cuijpers et al., 2020, 2021), prevalence rates remain high, highlighting the need for new treatment approaches. One treatment modality that offers promise is lifestyle therapy (e.g. targeting diet and physical activity; Firth et al., 2020; Wong et al., 2021). Evidence from clinical trials (Jacka et al., 2017; Parletta et al., 2019) and systematic reviews (Bizzozero-Peroni et al., 2024; Swainson et al., 2023) has demonstrated that dietary interventions can improve mental health symptoms in people with major and sub-threshold depression. Similar evidence from meta-analyses has also established physical activity as an effective way to reduce depressive symptoms (Noetel et al., 2024; Stubbs et al., 2016).

Notwithstanding the potential benefits, lifestyle interventions, like any form of treatment, can lead to differential outcomes depending on the population. For example, better social and cognitive function, higher baseline depression (Sotsky et al., 2006), older age and absence of physical comorbidities (Catarino et al., 2018) predict greater depressive symptom reduction in psychotherapy. On the other hand, chronic illness (Van et al., 2008), psychiatric comorbidity (Johnsen and Friborg, 2015) and a higher number of pre-trial safety events (i.e. negative medical or psychological events; Yacaman-Mendez et al., 2019) can predict poorer responses. In addition to influencing treatment outcomes, biopsychosocial markers similar to those above (i.e. physical quality of life (Croatto et al., 2022), social skills (You et al., 2024), blood markers and brain connectivity (Gotti et al., 2024) can also be modified through behavioural intervention via psychobiological mechanisms (Schotte et al., 2006; Suneson et al., 2021), highlighting a bidirectional relationship between these factors and treatment outcomes.

Because safety events occurring within pharmacological interventions are essential to outcome interpretation, trial governance and participant well-being, recording and reporting them is a regulatory requirement within such trials (Phillips et al., 2019; Vaughan et al., 2014). However, systematic reporting of these events in behavioural interventions has only recently become common (Klatte et al., 2023a; Papaioannou et al., 2021). As the field of lifestyle psychiatry develops, there have been calls for transparent reporting of safety events to clarify who benefits and who may be harmed by these types of behavioural interventions (O’Neil et al., 2025a). In addition, predictors of such events, and how the risk-benefit profile of lifestyle therapy compares to established therapies, such as psychotherapy, have not been previously examined.

Aiming to address these gaps in the literature, we conducted a secondary analysis of data from the recently completed Curbing Anxiety and depression using Lifestyle Medicine (CALM) randomised controlled trial (O’Neil et al., 2024). This trial used an individual randomised group-based design to compare the effects of lifestyle therapy to psychotherapy delivered over videoconferencing for managing mental health symptoms during the COVID-19 pandemic. CALM demonstrated, for the first time, that lifestyle therapy was non-inferior to psychotherapy for reducing depression over 8 weeks. In this secondary analysis, we aimed to identify psychological, biological and demographic factors that predicted benefits and safety events of the respective treatments during the trial. Noting findings in the main results paper, we also sought to examine safety events reported in the trial, to better understand the higher number of safety events reported in the lifestyle arm (*n* = 122) compared with psychotherapy arm (*n* = 79).

## Methods

A comprehensive account of the CALM trial has been published in the protocol (Young et al., 2022) and primary results paper (O’Neil et al., 2024). Briefly, 182 participants were recruited and enrolled through a mental health service and online recruitment in Victoria, Australia and randomised to either the group-based lifestyle or psychotherapy arm. Six 90-minute, group-based sessions were conducted over 8 weeks, delivered by two Psychologists (psychotherapy) or an Accredited Exercise Physiologist and Dietitian (lifestyle therapy) via Zoom. The lifestyle therapy programme was informed by previous models in mental health and diabetes prevention (see: Absetz et al., 2007; Jacka et al., 2017; Laatikainen et al., 2007; Lindström et al., 2013; Opie et al., 2018). The psychotherapy programme used a Cognitive Behaviour Therapy approach (Nathan et al., 2004), and mindfulness practices (Goldberg et al., 2018). Participants were provided workbooks and additional resources related to their treatment – a food hamper, a resistance band and a Fitbit (Fitbit, 2020) in the lifestyle arm, and self-soothing items in the psychotherapy arm.

### Participants

Participants were eligible for enrolment if they were: aged 18 or older; based in Victoria, Australia; able to provide written informed consent; able to attend the virtual group-based sessions; proficient in written English and basic digital literacy; and scored eight or higher on the Distress Questionnaire-5 (a commonly accepted broad screening tool used to identify likely cases of depression and anxiety; Batterham et al., 2016). Participants were excluded if they had: a clinically unstable medical disorder; current or past formally diagnosed eating disorder; severe dietary allergies, intolerances or aversions; socio-cultural, religious or medical reasons interfering with participation; enrolled in another trial; or were pregnant or planning to conceive or breastfeed. Participants could continue any pharmacological and psychological therapies during the trial, provided they did not commence a new or duplicative treatment in the 4 weeks prior to baseline. Full demographic information is included in the primary results paper (O’Neil et al., 2024).

### Measures

For the purpose of this paper, we were interested in exploring how the biological, demographic and psychological characteristics of participants upon trial entry (i.e. at baseline assessment) subsequently shaped their positive (benefits) and/or negative experiences (safety events) during treatment. Analyses were exploratory, and inclusion of exposure and outcome variables was reached by team consensus, based on prior literature. Demographic factors were included as they have repeatedly been shown to influence behavioural therapy outcomes (Bernal et al., 1998; Catarino et al., 2018; Joutsenniemi et al., 2012), along with initial symptom severity (Sotsky et al., 2006). Biological markers were also included as exposure variables due to recent work highlighting the importance of including them in psychological intervention studies, per the high comorbidity between mood disorders and chronic physical health conditions (Croatto et al., 2022). We additionally investigated if ‘responders’ (participants who achieved treatment ‘benefit’ (described in the Outcomes section) had a different likelihood of experiencing safety events compared to ‘non-responders’.

### Exposures

Baseline assessments were conducted via Computer-Assisted Telephone Interview by blinded research assistants. Psychological exposure variables collected during these interviews included the Patient Health Questionnaire (PHQ-9; Kroenke et al., 2001) to assess depressive symptoms and Generalised Anxiety Disorder questionnaire (GAD-7; Spitzer et al., 2006) to assess anxiety symptoms.

Biological markers were collected through fasting blood tests conducted after the baseline assessment and before the first intervention session, while demographic variables were collected via phone interview upon enrolment. Place of birth was coded as ‘born in Australia’ and ‘born outside of Australia’. See Table 1 for the specific variables.

**Table 1.** Summary of exposure variables assessed in the secondary CALM analysis.

Type	Exposure Variable
Demographic	Age
	Biological Sex
	Place of Birth
Psychological	Baseline PHQ-9
	Baseline GAD-7
Biological	Cholesterol
	Cholesterol:HDL ratio
	Glucose
	HDL
	LDL
	Triglycerides

### Outcomes

Our primary outcome was beneficial treatment response, defined as achieving at least a 50% reduction in PHQ-9 scores between baseline and 8-week follow-up, which is considered a clinically meaningful improvement regardless of initial severity (Coley et al., 2020). The PHQ-9 operationalises the nine DSM criteria for major depressive disorder, and has demonstrated strong psychometric properties (α = 0.892; test–retest reliability 0.737; Sun et al., 2020), including high external validity against the DSM-IV (sensitivity and specificity = 88%; Kroenke et al., 2001). The scale’s accuracy and sensitivity (Levis et al., 2019; Manea et al., 2015) make it particularly suitable for measuring treatment response. Because there are various ways to determine improvement in psychological symptoms, we also used other metrics and measures of improvement as supplementary outcomes, using the PHQ-9 and also the GAD-7 for anxiety. The GAD-7 has excellent internal consistency (α = 0.89-92; Zhong et al., 2015), test–retest reliability (ICC = 0.83) and external validity against the DSM-IV (sensitivity = 89%, specificity = 82%) and is widely used for measuring changes in anxiety symptoms (Spitzer et al., 2006). Using these two scales, the following exploratory outcomes which used different definitions of benefit were included to decrease any bias that may have occurred due to outcome measure type, and are included in supplementary materials: (1) reliable change in PHQ-9 and GAD-7 at 8 weeks, calculated using a reference standard deviation from the Australian population during COVID-19 (6.0 for PHQ-9 and 5.3 for GAD-7; Stocker et al., 2021), a conservative Cronbach’s alpha of 0.75, and using the cut-off score of 1.96 (Jacobson et al., 1991); (2) PHQ-9 remission, defined as a score of < 5 at 8 weeks, and (3) 50% reduction in GAD-7 at 8 weeks.

Safety events were safety events (secondary outcome), defined as any untoward medical occurrence, unintended disease or injury or untoward signs (including abnormal laboratory findings of clinical significance) experienced by participants that could be deemed as either related or unrelated to the study intervention. They included the worsening or recurrence of a pre-existing condition, but did not include any medical occurrence that began prior to intervention. While harm could be defined in numerous ways (e.g. deterioration on the primary outcome, which was infrequent (11.5%; see Table 2), these safety events were selected to allow greater exploration of the range of safety events experienced, and their relevance to the trial or treatment was adjudicated by a medical professional within the trial team.

**Table 2.** Demographics and benefit/safety event outcomes by treatment arm in the per-protocol sample of CALM trial participants.

	Lifestyle Therapy (*N* = 71)	Psychotherapy (*N* = 64)	Total (*N* = 135)
Participant Age	46.6 (14.4)	43.9 (13.8)	45.3 (14.2)
% Female	66 (93.0%)	49 (76.6%)	115 (85.2%)
SEIFA-IRSD^$^	1012.7 (54.2)	1029.2 (45.8)	1020.3 (50.8)
% Employed	53 (74.6%)	45 (70.3%)	98 (72.6%)
% Completed high school	61 (85.9%)	59 (92.2%)	120 (88.9%)
% Born in Australia	61 (85.9%)	50 (79.4%)	111 (82.8%)
Total number of sessions attended	5.2 (0.8)	5.0 (1.0)	5.1 (0.9)
Cholesterol (mmol/L)	5.2 (0.9)	5.5 (1.0)	5.3 (1.0)
HDL^%^ (mmol/L)	1.8 (0.5)	1.7 (0.4)	1.7 (0.4)
LDL^§^ (mmol/L)	2.9 (0.8)	3.3 (0.9)	3.1 (0.8)
Triglycerides (mmol/L)	1.2 (0.6)	1.2 (0.8)	1.2 (0.7)
Total Cholesterol:HDL	3.2 (1.1)	3.5 (1.0)	3.3 (1.0)
Glucose (mmol/L)	4.9 (0.8)	4.9 (0.6)	4.9 (0.7)
Total PHQ-9 at Baseline	10.1 (6.1)	10.4 (5.4)	10.3 (5.8)
Total GAD-7 at Baseline	9.1 (5.2)	8.7 (4.3)	8.9 (4.8)
*PHQ-9 50% Change (benefit)*			
Deterioration	8 (11.6%)	7 (11.3%)	15 (11.5%)
No Change	32 (46.4%)	34 (54.8%)	66 (50.4%)
Improvement	29 (42.0%)	21 (33.9%)	50 (38.2%)
*Safety Events*			
Average number of safety events during treatment	1.72 (1.39)	1.23 (0.92)	1.49 (1.21)
Total number of safety events	122	79	201
Total safety events related to the intervention	4	0	4
% of participants reporting safety events	55 (78.6%)	48 (77.4%)	103 (78.0%)

$ Socio-economic indexes for areas- index of relative socio-economic disadvantage; ^%^high-density lipoprotein cholesterol; ^§^low density lipoprotein cholesterol.

While safety events were captured from baseline assessment, for the purpose of this study, we included only those from the first session onwards as we were interested in the potential of the safety events being associated with the treatments. Safety events were primarily collected via fortnightly self-report questionnaires from the first session onwards. Participants were asked to describe any unexpected changes in their mood and physical health. Follow-up questions included: date of onset, if symptoms were still impacting them, hospitalisation or any other medical attention sought, the severity of the symptoms (mild, moderate, severe), and if the participant believed the change was related to their participation in the trial. In addition, safety events were also monitored through a non-systematic assessment including use of clinical observation during intervention sessions or assessments; clinical pathology at follow-up; and spontaneous self-report in communications with trial staff. All events were classified using Medical Dictionary for Regulatory Activities (MedDRA) standardised medical terminology and coding (Morley, 2014). This comprehensive approach to safety monitoring aligns with recent recommendations for behavioural intervention trials, where traditional adverse event frameworks may not fully capture the range of participant experiences, particularly in the context of treatment for symptoms of mental ill-health (Taher et al., 2023).

### Statistical analysis

We fitted Generalised Estimating Equations (GEE) regression models with robust Huber Sandwich estimators to account for the clustering attributable to group blocks. For the binary treatment response outcomes (50% reduction in PHQ-9, along with supplementary outcomes of 50% reduction in GAD-7; reliable change in PHQ-9 and GAD-7; and PHQ-9 remission), we fitted Poisson GEE regression with a log link function and exponentiated the results to estimate risk ratios. For safety event outcomes, we used Poisson GEE regression with a log link function (un-exponentiated) to estimate expected counts. We conducted two models to assess the relationship between each predictor and outcome in (1) the overall sample (primary aim) and (2) separately for psychotherapy and lifestyle therapy (exploratory aim; provided in supplementary materials). Model assumptions were assessed prior to model fitting. We included both per-protocol (PP) and intention-to-treat (ITT) analyses, whereby we imputed missing data using multiple imputation chained equations. As ITT can bias findings in non-inferiority trials to erroneously conclude non-inferiority (D’Agostino et al., 2003), unless otherwise specified, results reported below are based on PP analysis, with inconsistencies relative to ITT analysis addressed. We used Simes adjusted *p*-values, termed q-values, to account for multiple testing (Simes, 1986). The Simes method adjusts the *p*-value, not the inference threshold and so q-values are interpreted in the same manner as a traditional *p*-value. Q-values were calculated separately for each outcome, subgroup (full sample, psychotherapy and lifestyle therapy) and model (PP and ITT). All analyses were performed using Stata version 18.0.

While the CALM trial had a satisfactory sample size, many of the secondary analyses conducted here are underpowered, making the results, especially those conducted by arm, exploratory.

## Results

### Sample characteristics

Table 2 shows the characteristics of those included in the present analysis and the distribution of benefits and safety events experienced. A total of 135 of the randomised 182 participants completed the CALM trial. For per-protocol results for PHQ-9 only, the included sample was *n* = 132 due to missing data on the primary outcome; the per-protocol sample size for all other measures was *n* = 135. By chance, more participants in the lifestyle therapy arm were female and had lower baseline LDL cholesterol. There were no differences between treatment arms on any other measure.

### Trial benefits

Overall, 38% of participants experienced benefit from treatment according to our primary outcome criteria (see Table 2). In the lifestyle therapy arm, 42.0% experienced benefit, and in the psychotherapy arm, 33.9% experienced benefit. There were no between-arm differences for treatment benefit (RR: 1.22, 95% CI [0.84, 1.78]).

**Figure 1.**
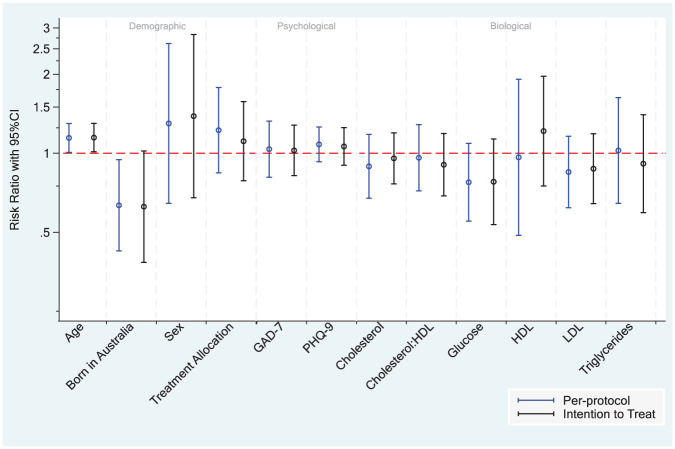
Association of baseline predictors with risk of 50% reduction in PHQ-9 scores from baseline to 8-week follow-up for the whole sample. Age–per 10-year increase in age; Born in Australia–reference group = born overseas; Cholesterol, HDL, LDL, Triglycerides, Glucose–per mmol/L increase; GAD-7–per 5 unit increase in baseline GAD-7 score; PHQ-9–per 5 unit increase in baseline PHQ-9; Sex–reference group = males; Total Cholesterol:HDL ratio of total cholesterol (mmol/L) to HDL (mmol/L); Treatment Arm–reference group = psychotherapy. 95% CI = 95% Confidence Interval; GAD-7 = 7-item generalised anxiety disorder scale; HDL = high-density lipoprotein; LDL = low density lipoprotein; PHQ-9 = 9-item patient health questionnaire.

For the overall sample, participants who were older (RR: 1.14 per 10-year increase in age, 95% CI [1.01, 1.30]) were more likely to fulfil criteria for treatment benefit (Figure 1). In contrast, participants who were born in Australia were less likely to achieve treatment benefit (RR: 0.63, 95% CI [0.42 to 0.94]). No other psychological, biological or demographic variables were associated with increased likelihood of benefitting from treatment. These results held true for age when re-analysed using the ITT, and while they were of similar magnitude for place of birth, only weak evidence was demonstrated.

In psychotherapy, older participants were more likely to achieve benefit from treatment, whereas those with higher baseline LDL and cholesterol:HDL ratio were less likely to achieve benefit. The age effect held in ITT analysis, but the lipid effects did not, although they were directionally consistent. In the lifestyle arm, higher baseline glucose and being born in Australia predicted lower likelihood of benefit, with ITT analyses yielding consistent findings except that baseline glucose was no longer significant. Full subgroup results are available in Supplementary Materials (see Figure 2), in addition to supplementary outcomes for predictors of PHQ-9 reliable change and remission, along with GAD-7 reliable change and 50% reduction in the overall sample.

### Safety events

In the overall sample, 78% of participants experienced at least one safety event (mean: 1.49 per participant). Rates by treatment were comparable; 78.6% and 77.4% of those in the lifestyle and psychotherapy arms reported at least one safety event, respectively (1.72 vs 1.23 per participant; Table 2).

In terms of the type and nature of safety events experienced, a total of 201 safety events were reported during the treatment period (122 in the lifestyle arm and 79 in the psychotherapy arm; Table 3). Those in the lifestyle arm reported more total safety events than those in psychotherapy (β: 0.47, 95% CI [0.16, 0.78]). The most common events were distress (29.9% of all events), hypercholesterolemia (20.4%), low mood (16.9%) and upper respiratory tract infection (5.0%), with similar profiles of events between arms. There was also a higher number of safety events reported by participants who did not achieve trial benefit (50% reduction on the PHQ-9; trial ‘non-responders’) compared to those who did (trial responders; 129 vs 72; *p* < 0.001), with the same types of events being most common. See Supplementary Materials for the table of all safety event subtypes.

**Table 3.** Safety events for the overall sample, by treatment arm and by treatment response profile (responders vs non-responders).

	Overall	Psychotherapy	Lifestyle Therapy	*p*-value	Non-Responders	Responders	*p*-value
***Total***	***201***	***79***	***122***	***0.002***	***129***	***72***	***<*** ***0.001***
Mental Health Related	**97 (48.3%)**	38 (48.1%)	59 (48.4%)	0.033	64 (49.6%)	33 (45.8%)	0.002
Cardiometabolic Related	**49 (24.4%)**	17 (21.5%)	32 (26.2%)	0.032	34 (26.4%)	15 (20.8%)	0.007
Pain, Injury, Inflammation, Musculoskeletal related	**20 (10.0%)**	9 (11.4%)	11 (9.0%)	0.655	13 (10.1%)	7 (9.7%)	0.180
Illness or Allergy Related	**19 (9.5%)**	6 (7.6%)	13 (10.7%)	0.108	8 (6.2%)	11 (15.3%)	0.491
Gastrointestinal Related	**10 (5.0%)**	6 (7.6%)	4 (3.3%)	0.527	8 (6.2%)	2 (2.8%)	0.058
Other	**6 (3.0%)**	3 (3.8%)	3 (2.5%)	1.000	2 (1.6%)	4 (5.6%)	0.414

For the overall sample, every 10-year increase in age was associated with a 0.16 higher number of reported safety events (95% CI [0.05, 0.27]). In addition, higher baseline glucose (β: 0.16 per mmol/L increase in glucose, 95% CI [0.10, 0.23]) was also associated with a higher number of reported safety events. ITT results were matching in magnitude and direction.

By treatment arm, older age was only associated with more safety events in the lifestyle arm. Higher baseline glucose was associated with more safety events in both arms, although this did not hold true in the psychotherapy arm for ITT analysis. No other predictors were found to be related to safety event outcomes.

## Discussion

In this secondary analysis of the CALM trial, we observed an overall treatment response rate of 38%, consistent with other psychosocial interventions for depression (Cuijpers et al., 2021), underscoring the ongoing challenge of achieving robust benefit for all participants in real-world samples with elevated distress. The proportion of participants reporting at least one safety event (78%) was substantially higher than most prior reports from behavioural intervention trials (e.g. 52.6% in psychotherapeutic trials for depression (Moritz et al., 2019). However, these rates are often poorly measured and/or under-reported (Klatte et al., 2023b; O’Neil et al., 2025a, 2025b), making this discrepancy likely due to rigorous and systematic harm assessment in this trial. Our approach aligns with recent recommendations that call for more structured and comprehensive adverse event monitoring in psychological and lifestyle interventions (Klatte et al., 2023a; O’Neil, 2025a).

While the proportion of participants experiencing at least one safety event was similar across treatment arms, there was a higher number of safety events reported per participant in the lifestyle arm than in the psychotherapy arm. This may reflect the broader range of activities within lifestyle interventions, including modifications to diet, physical activity and other health behaviours. As individuals adjusted to new routines or physical demands, this could have led to an increased likelihood of physical health fluctuations and minor injuries or side effects (Niemeijer et al., 2020; O’Dougherty et al., 2012), or heightened vigilance and reporting of bodily changes (Berthelot et al., 2019).

However, the types of safety events experienced were similar between arms, and only four of 201 safety events were deemed related to the intervention. This finding echoes the distinction made in the literature between events that occur during treatment and those that are causally linked to treatment (Klatte et al., 2018). It deems risk to be comparable between arms, reinforcing the safety of lifestyle interventions when delivered with proper monitoring (Marx et al., 2023). These unrelated safety events may reflect background risk, including underlying conditions or external stressors, rather than direct intervention effects. The prevalence of psychological safety events such as distress or low mood is common in mental health populations and can often be transient or arise through engagement with difficult material (Vybíral et al., 2024), while physical events (e.g. hypercholesterolemia, infections) likely reflect comprehensive reporting rather than causal effects. Finally, people who benefitted from treatment reported fewer safety events than those who did not. This pattern may reflect that those who did not benefit from the intervention remained more vulnerable to harms, or that those experiencing fewer safety events were more likely to persist with prescribed behaviours and benefit from the intervention. This is a relatively underexplored area in the literature and merits further investigation.

Across arms, older age and being born outside of Australia were predictive of treatment benefit, while older age and elevated baseline blood glucose levels were predictive of safety events. All results are preliminary, and exploratory subgroup analyses were particularly underpowered and should be interpreted with caution. Results pertinent to the overall sample are discussed below, highlighting variables that may be relevant to clinical decision-making, guiding the selection of consumers who may most benefit from lifestyle-based interventions, whether as alternatives or adjuncts to standard mental healthcare. In addition to these variables, it is also important to consider for which individuals the systemic effects of lifestyle interventions (i.e. physical health, sleep) may be the most beneficial for, beyond effectively reducing depressive symptoms (e.g. older individuals or those with chronic health conditions).

As age increased, participants in the overall sample and psychotherapy arm were more likely to achieve benefit, consistent with prior findings that higher age is associated with greater gains in cognitive and psychological interventions (Catarino et al., 2018; Cuijpers et al., 2022). However, in the lifestyle arm, benefit was consistent across the age spectrum, suggesting that lifestyle interventions may be equally effective regardless of age. Given that older age was also related to a greater number of reported safety events in the lifestyle arm, psychotherapy could be better suited to older individuals from a risk-benefit perspective.

Place of birth was also a key predictor, requiring further investigation: participants born outside of Australia were more likely to experience benefit in the overall sample and lifestyle arm, while this effect was not seen in psychotherapy. As this variable likely functions as a proxy for other factors (i.e. socio-economic status, acculturation), further research is needed to understand which aspects are most relevant to outcomes. Research on cultural and migration variables as predictors of treatment response is limited by sample homogeneity (Thielecke et al., 2024), although one study suggests that people with migrant backgrounds may respond particularly well to psychosocial interventions, accounted for by higher levels of baseline post-traumatic stress symptoms and interpersonal problems that are alleviated by the intervention (Wiborg et al., 2016). Nevertheless, as the group-based format was consistent across arms in this study, it is less likely that alleviation of interpersonal stress due to group connection alone explained the benefit for participants born outside of Australia. Instead, although the CALM programme was not culturally adapted, aspects of the lifestyle intervention (such as Mediterranean dietary suggestions) may have been more culturally congruent or appealing to those born outside Australia. Culturally tailored interventions can indeed improve engagement and outcomes across mental health settings (Bernal and Domenech Rodríguez, 2012), and our findings highlight the importance of considering individuals’ backgrounds when implementing future lifestyle therapies, and highlight the need for further culturally informed research in diverse and multicultural samples.

Finally, elevated baseline glucose and older age were associated with a greater number of reported safety events. The latter was observed only in the lifestyle arm, where the overall number of reported safety events was also higher than in psychotherapy. Though these results could indicate that individuals with these characteristics had poorer health and therefore experienced more frequent harms, other factors may be influencing the association. Older age and higher blood glucose could reflect underlying vulnerabilities or chronic health conditions that may increase risk during the intervention, or simply lead to a greater likelihood of reporting health concerns. Furthermore, in the lifestyle arm, participants and interventionsists may have been more vigilant about monitoring those of older age and/or with elevated glucose (and likely other comorbidities) due to the influence of diet and exercise on these outcomes (Sevild et al., 2020).

### Limitations

Our sample was of modest size comprising predominantly middle-aged, educated, Anglo-Celtic women born in Australia, consistent with bias typically found in clinical trial populations (Hughson et al., 2016; Wang et al., 2007). This may have limited the generalisability of findings and constrained the examination of demographic predictors. Both PP and ITT analyses were conducted and generally yielded convergent results, although where discrepancies occurred, significance was only in the PP sample, likely reflecting the missing data. Since the PP model was primarily reported, the sample was restricted to those who completed the 8-week assessment, which may bias results towards motivated individuals more likely to be experiencing benefit. Reasons for dropout are discussed in the main results paper, and are typical of a remote-delivered lifestyle intervention in a mental health population (O’Neil et al., 2024). Power for subgroup analyses was limited, so treatment specific predictors should be interpreted cautiously and emphasis placed on predictors observed in the full sample for the primary benefit and secondary safety events outcome, as they may explain response mechanisms common in both types of behavioural mental health interventions. In addition, only a minority of all reported safety events were adjudicated as directly related to the intervention, which aligns with the reality that many safety events in behavioural trials are difficult to attribute definitively (Ioannidis et al., 2004). Including all reported safety events in the analysis remains justified, as it provides a comprehensive view of participant safety and vulnerability, even if causality cannot be fully established.

### Implications and future research

Mental health treatments, including lifestyle interventions, are not universally effective and are not without risk. Only a minority of individuals achieve meaningful improvement (Cuijpers et al., 2021), and identifying who may experience this response is critical. Our findings underscore the value of considering cultural background and age effects when personalising care, and further investigating participant characteristics in relation to treatment success. For example, older individuals seemed to benefit from psychotherapy while being more likely to experience harm from the lifestyle intervention. Though opportunities for personalised treatment are a privilege, high-quality evidence of clinically significant benefit and harm predictors, could increase motivation for increasing consumer autonomy in their treatment plan. Future research should replicate these analyses in larger, more diverse samples to improve predictive power and generalisability. Ensuring demographic variability and accounting for cultural influences is critical for designing equitable and effective interventions. Studies need to explore long-term outcomes and further examine the predictors and mechanisms underlying sustained benefit and safety in lifestyle-based mental health care.

## Conclusion

Across the CALM trial treatment arms, older age and being born outside of Australia were predictive of treatment benefit, while older age and elevated baseline blood glucose levels predicted a greater number of safety events. Personalising lifestyle therapies based on individual characteristics such as age and place of birth (including socio-economic status and cultural beliefs) may enhance their effectiveness while minimising risk. Our results highlight the need to focus future investigations on developing individualised, culturally responsive mental health interventions, in order to refine treatment selection and improve outcomes for diverse populations.

## Supplemental Material

sj-xlsx-1-anp-10.1177_00048674261440324 – Supplemental material for Predictors of benefits and safety events from participation in a lifestyle therapy trial for adults with depression: A secondary analysis of the CALM randomised controlled trial Supplemental material, sj-xlsx-1-anp-10.1177_00048674261440324 for Predictors of benefits and safety events from participation in a lifestyle therapy trial for adults with depression: A secondary analysis of the CALM randomised controlled trial by Lara K Radovic, Madeleine L Connolly, Lauren M Young, Marita Bryan, Wolfgang Marx, Megan Turner, Dean Saunders, Sophie Mahoney, Rachel Fiddes, Tayla John, Amelia McGuinness, Tetyana Rocks, Deborah N Ashtree and Adrienne O’Neil in Australian & New Zealand Journal of Psychiatry

## References

[bibr1-00048674261440324] Absetz P Valve R Oldenburg B , et al. (2007) Type 2 diabetes prevention in the real world: One-year results of the GOAL implementation trial. Diabetes Care 30: 2465–2470. 17586741 10.2337/dc07-0171

[bibr2-00048674261440324] Batterham PJ Sunderland M Carragher N , et al. (2016) The distress questionnaire-5: Population screener for psychological distress was more accurate than the K6/K10. Journal of Clinical Epidemiology 71: 35–42. 26464194 10.1016/j.jclinepi.2015.10.005

[bibr3-00048674261440324] Bernal G Domenech Rodríguez MM (2012) Cultural Adaptations: Tools for Evidence-Based Practice with Diverse Populations. Washington, DC: American Psychological Association.

[bibr4-00048674261440324] Bernal G Bonilla J Padilla-Cotto L , et al. (1998) Factors associated to outcome in psychotherapy: An effectiveness study in Puerto Rico. Journal of Clinical Psychology 54: 329–342. 9545170 10.1002/(sici)1097-4679(199804)54:3<329::aid-jclp4>3.0.co;2-k

[bibr5-00048674261440324] Berthelot JM Nizard J Maugars Y (2019) The negative Hawthorne effect: Explaining pain overexpression. Joint Bone Spine 86: 445–449. 30316973 10.1016/j.jbspin.2018.10.003

[bibr6-00048674261440324] Bizzozero-Peroni B Martínez-Vizcaíno V Fernández-Rodríguez R , et al. (2024) The impact of the Mediterranean diet on alleviating depressive symptoms in adults: A systematic review and meta-analysis of randomised controlled trials. Nutrition Reviews 83: 29–39. 10.1093/nutrit/nuad176 38219230

[bibr7-00048674261440324] Catarino A Bateup S Tablan V , et al. (2018) Demographic and clinical predictors of response to internet-enabled cognitive–behavioral therapy for depression and anxiety. BJPsych Open 4: 411–418. 30294451 10.1192/bjo.2018.57 PMC6171334

[bibr8-00048674261440324] Coley RY Boggs JM Beck A , et al. (2020) Defining success in measurement-based care for depression: A comparison of common metrics. Psychiatric Services 71: 312–318. 31847739 10.1176/appi.ps.201900295

[bibr9-00048674261440324] Correll CU Solmi M Cortese S , et al. (2023) The future of psychopharmacology: A critical appraisal of ongoing phase 2/3 trials, and of some current trends aiming to de-risk trial programmes of novel agents. World Psychiatry 22: 48–74. 36640403 10.1002/wps.21056 PMC9840514

[bibr10-00048674261440324] Croatto G Vancampfort D Miola A , et al. (2022) The impact of pharmacological and non-pharmacological interventions on physical health outcomes in people with mood disorders across the lifespan: An umbrella review of the evidence from randomised controlled trials. Molecular Psychiatry 28: 369–390. 36138129 10.1038/s41380-022-01770-w PMC9493151

[bibr11-00048674261440324] Cuijpers P Ciharova M Quero S , et al. (2022) The contribution of ‘individual participant data’ meta-analyses of psychotherapies for depression to the development of personalised treatments: A systematic review. Journal of Personalised Medicine 12: 93. 10.3390/jpm12010093 PMC8781368 35055408

[bibr12-00048674261440324] Cuijpers P Karyotaki E Ciharova M , et al. (2021) The effects of psychotherapies for depression on response, remission, reliable change, and deterioration: A meta-analysis. Acta Psychiatrica Scandinavica 144: 288–299. 34107050 10.1111/acps.13335 PMC8457213

[bibr13-00048674261440324] Cuijpers P Noma H Karyotaki E , et al. (2020) A network meta-analysis of the effects of psychotherapies, pharmacotherapies and their combination in the treatment of adult depression. World Psychiatry 19: 92–107. 31922679 10.1002/wps.20701 PMC6953550

[bibr14-00048674261440324] D’Agostino RB Sr Massaro JM Sullivan LM (2003) Non-inferiority trials: Design concepts and issues–the encounters of academic consultants in statistics. Statistics in Medicine 22: 169–186. 12520555 10.1002/sim.1425

[bibr15-00048674261440324] Firth J Solmi M Wootton RE , et al. (2020) A meta-review of ‘lifestyle psychiatry’: The role of exercise, smoking, diet and sleep in the prevention and treatment of mental disorders. World Psychiatry 19: 360–380. 32931092 10.1002/wps.20773 PMC7491615

[bibr16-00048674261440324] Fitbit (2020) Fitbit charge 4. Available at: www.fitbit.com/global/au/products/trackers/charge4 (accessed November 4, 2025).

[bibr17-00048674261440324] Goldberg SB Tucker RP Greene PA , et al. (2018) Mindfulness-based interventions for psychiatric disorders: A systematic review and meta-analysis. Clinical Psychology Review 59: 52–60. 29126747 10.1016/j.cpr.2017.10.011 PMC5741505

[bibr18-00048674261440324] Gotti G Gabelli C Russotto S , et al. (2024) Biomarkers of response to internet-based psychological interventions: Systematic review. Journal of Medical Internet Research 26: e55736. 10.2196/55736 PMC11645513 39612489

[bibr19-00048674261440324] Hughson JA Woodward-Kron R Parker A , et al. (2016) A review of approaches to improve participation of culturally and linguistically diverse populations in clinical trials. Trials 17: 263. 27229153 10.1186/s13063-016-1384-3 PMC4880985

[bibr20-00048674261440324] Ioannidis JPA Evans SJW Gøtzsche PC , et al. (2004) Better reporting of harms in randomised trials: An extension of the CONSORT statement. Annals of Internal Medicine 141: 781–788. 15545678 10.7326/0003-4819-141-10-200411160-00009

[bibr21-00048674261440324] Jacka FN O’Neil A Opie R , et al. (2017) A randomised controlled trial of dietary improvement for adults with major depression (the ‘SMILES’ trial). BMC Medicine 15: 23. 28137247 10.1186/s12916-017-0791-y PMC5282719

[bibr22-00048674261440324] Jacobson NS Follette WC Revenstorf D (1991) Toward a standard definition of clinically significant change. Psychotherapy Research 1: 16–26.

[bibr23-00048674261440324] Johnsen TJ Friborg O (2015) The effects of cognitive behavioral therapy as an anti-depressive treatment is falling: A meta-analysis. Psychological Bulletin 141: 747–768. 25961373 10.1037/bul0000015

[bibr24-00048674261440324] Joutsenniemi K Laaksonen MA Knekt P , et al. (2012) Prediction of the outcome of short- and long-term psychotherapy based on socio-demographic factors. Journal of Affective Disorders 141: 331–342. 22520738 10.1016/j.jad.2012.03.027

[bibr25-00048674261440324] Klatte R Strauss B Flückiger C , et al. (2018) Adverse effects of psychotherapy: Protocol for a systematic review and meta-analysis. Systematic Reviews 7: 135. 30193585 10.1186/s13643-018-0802-x PMC6128985

[bibr26-00048674261440324] Klatte R Strauss B Flückiger C , et al. (2023a) Adverse events in psychotherapy randomised controlled trials: A systematic review. Psychotherapy Research 35: 84–99. 38090772 10.1080/10503307.2023.2286992

[bibr27-00048674261440324] Klatte R Strauss B Flückiger C , et al. (2023b) Defining and assessing adverse events and harmful effects in psychotherapy study protocols: A systematic review. Psychotherapy 60: 130–148. 35049321 10.1037/pst0000359

[bibr28-00048674261440324] Kroenke K Spitzer RL Williams JB (2001) The PHQ-9: Validity of a brief depression severity measure. Journal of General Internal Medicine 16: 606–613. 11556941 10.1046/j.1525-1497.2001.016009606.x PMC1495268

[bibr29-00048674261440324] Laatikainen T Dunbar JA Chapman A , et al. (2007) Prevention of type 2 diabetes by lifestyle intervention in an Australian primary health care setting: Greater Green Triangle (GGT) diabetes prevention project. BMC Public Health 7: 1–7. 17877832 10.1186/1471-2458-7-249 PMC2039742

[bibr30-00048674261440324] Levis B Benedetti A Thombs BD (2019) Accuracy of patient health questionnaire-9 (PHQ-9) for screening to detect major depression: Individual participant data meta-analysis. BMJ 365: 1476. 10.1136/bmj.l1476 PMC6454318 30967483

[bibr31-00048674261440324] Lindström J Peltonen M Eriksson JG , et al. (2013) Improved lifestyle and decreased diabetes risk over 13 years: Long-term follow-up of the randomised Finnish Diabetes Prevention Study (DPS). Diabetologia 56: 284–293. 23093136 10.1007/s00125-012-2752-5

[bibr32-00048674261440324] Manea L Gilbody S McMillan D (2015) A diagnostic meta-analysis of the patient health questionnaire-9 (PHQ-9) algorithm scoring method as a screen for depression. General Hospital Psychiatry 37: 67–75. 25439733 10.1016/j.genhosppsych.2014.09.009

[bibr33-00048674261440324] Marx W Manger SH Blencowe M , et al. (2023) Clinical guidelines for the use of lifestyle-based mental health care in major depressive disorder: World federation of societies for biological psychiatry (WFSBP) and Australasian society of lifestyle medicine (ASLM) taskforce. The World Journal of Biological Psychiatry 24: 333–386. 36202135 10.1080/15622975.2022.2112074 PMC10972571

[bibr34-00048674261440324] Moritz S Nestoriuc Y Rief W , et al. (2019) ‘It can’t hurt, right?’ Adverse effects of psychotherapy in patients with depression. European Archives of Psychiatry and Clinical Neuroscience 269: 577–586. 30088072 10.1007/s00406-018-0931-1

[bibr35-00048674261440324] Morley G (2014) Adverse event reporting: A brief overview of Meddra. Medical Writing 23: 113–116.

[bibr36-00048674261440324] Nathan PR Smith L Rees CS (2004) Mood Management Course: A Cognitive Behavioral Group Treatment Programme for Anxiety Disorders and Depression, 2nd Edition. Perth, WA, Australia: Centre for Clinical Interventions.

[bibr37-00048674261440324] Niemeijer A Lund H Stafne SN , et al. (2020) Adverse events of exercise therapy in randomised controlled trials: A systematic review and meta-analysis. British Journal of Sports Medicine 54: 1073–1080. 31563884 10.1136/bjsports-2018-100461

[bibr38-00048674261440324] Noetel M Sanders T Gallardo-Gómez D , et al. (2024) Effect of exercise for depression: Systematic review and network meta-analysis of randomised controlled trials. BMJ 384: e075847. 10.1136/bmj-2023-075847 PMC10870815 38355154

[bibr39-00048674261440324] O’Dougherty M Hearst MO Syed M , et al. (2012) Life events, perceived stress and depressive symptoms in a physical activity intervention with young adult women. Mental Health and Physical Activity 5: 148–154. 23189088 10.1016/j.mhpa.2012.05.001 PMC3505451

[bibr40-00048674261440324] O’Neil A John T Turner A , et al. (2025a) Advancing the quality and safety of behavioural interventions in mental health research: A how-to guide from the magnet clinical trial network. Australian and New Zealand Journal of Psychiatry 59: 315–321. 39991899 10.1177/00048674251319680 PMC11924282

[bibr41-00048674261440324] O’Neil A Perez J Young LM , et al. (2024) Clinical and cost-effectiveness of remote-delivered, online lifestyle therapy versus psychotherapy for reducing depression: Results from the CALM non-inferiority, randomised trial. The Lancet Regional Health – Western Pacific 49: 101142. 39381019 10.1016/j.lanwpc.2024.101142 PMC11459004

[bibr42-00048674261440324] O’Neil A Rossell SL Berk M (2025b) Understanding side effects of psychotherapies: Implications for clinical practice and research trials. World Psychiatry 24: 194–195. 40371754 10.1002/wps.21304 PMC12079470

[bibr43-00048674261440324] Opie RS O’Neil A Jacka FN , et al. (2018) A modified Mediterranean dietary intervention for adults with major depression: Dietary protocol and feasibility data from the SMILES trial. Nutritional Neuroscience 21: 487–501. 28424045 10.1080/1028415X.2017.1312841

[bibr44-00048674261440324] Papaioannou D Cooper C Mooney C , et al. (2021) Adverse event recording failed to reflect potential harms: A review of trial protocols of behavioral, lifestyle and psychological therapy interventions. Journal of Clinical Epidemiology 136: 64–76. 33684508 10.1016/j.jclinepi.2021.03.002

[bibr45-00048674261440324] Parletta N Zarnowiecki D Cho J , et al. (2019) A Mediterranean-style dietary intervention supplemented with fish oil improves diet quality and mental health in people with depression: A randomised controlled trial (HELFIMED). Nutritional Neuroscience 22: 474–487. 29215971 10.1080/1028415X.2017.1411320

[bibr46-00048674261440324] Phillips R Hazell L Sauzet O , et al. (2019) Analysis and reporting of adverse events in randomised controlled trials: A review. BMJ Open 9: 24537. 10.1136/bmjopen-2018-024537 PMC6398660 30826796

[bibr47-00048674261440324] Schotte CK Van Den Bossche B De Doncker D , et al. (2006) A biopsychosocial model as a guide for psychoeducation and treatment of depression. Depression and Anxiety 23: 312–324. 16688730 10.1002/da.20177

[bibr48-00048674261440324] Sevild CH Niemiec CP Bru LE , et al. (2020) Initiation and maintenance of lifestyle changes among participants in a healthy life centre: A qualitative study. BMC Public Health 20: 1006. 10.1186/s12889-020-09111-8 PMC7318496 32586299

[bibr49-00048674261440324] Simes RJ (1986) An improved Bonferroni procedure for multiple tests of significance. Biometrika 73: 751–754.

[bibr50-00048674261440324] Sotsky SM Glass DR Shea MT , et al. (2006) Patient predictors of response to psychotherapy and pharmacotherapy: Findings in the NIMH treatment of depression collaborative research program. Focus 4: 278–290. 10.1176/ajp.148.8.997 1853989

[bibr51-00048674261440324] Spitzer RL Kroenke K Williams JB , et al. (2006) A brief measure for assessing generalised anxiety disorder. Archives of Internal Medicine 166: 1092. 16717171 10.1001/archinte.166.10.1092

[bibr52-00048674261440324] Stocker R Tran T Hammarberg K , et al. (2021) Patient health questionnaire 9 (PHQ-9) and general anxiety disorder 7 (GAD-7) data contributed by 13,829 respondents to a national survey about COVID-19 restrictions in Australia. Psychiatry Research 298: 113792. 33592399 10.1016/j.psychres.2021.113792 PMC9754708

[bibr53-00048674261440324] Stubbs B Vancampfort D Rosenbaum S , et al. (2016) Challenges establishing the efficacy of exercise as an antidepressant treatment: A systematic review and meta-analysis of control group responses in exercise randomised controlled trials. Sports Medicine 46: 699–713. 26707338 10.1007/s40279-015-0441-5

[bibr54-00048674261440324] Sun Y Fu Z Bo Q , et al. (2020) The reliability and validity of PHQ-9 in patients with major depressive disorder in psychiatric hospital. BMC Psychiatry 20: 474. 32993604 10.1186/s12888-020-02885-6 PMC7525967

[bibr55-00048674261440324] Suneson K Lindahl J Chamli Hårsmar S , et al. (2021) Inflammatory depression – Mechanisms and non-pharmacological interventions. International Journal of Molecular Sciences 22: 1640. 33561973 10.3390/ijms22041640 PMC7915869

[bibr56-00048674261440324] Swainson J Reeson M Malik U , et al. (2023) Diet and depression: A systematic review of whole dietary interventions as treatment in patients with depression. Journal of Affective Disorders 327: 270–278. 36738997 10.1016/j.jad.2023.01.094

[bibr57-00048674261440324] Taher R Hsu CW Hampshire C , et al. (2023) The safety of digital mental health interventions: Systematic review and recommendations. JMIR Mental Health 10: e47433. 10.2196/47433 PMC10594135 37812471

[bibr58-00048674261440324] Thielecke J Kuper P Lehr D , et al. (2024) Who benefits from indirect prevention and treatment of depression using an online intervention for insomnia? Results from an individual-participant data meta-analysis. Psychological Medicine 54: 2389–2402. 38469832 10.1017/S0033291724000527

[bibr59-00048674261440324] Van HL Schoevers RA Dekker J (2008) Predicting the outcome of antidepressants and psychotherapy for depression: A qualitative, systematic review. Harvard Review of Psychiatry 16: 225–234. 18661365 10.1080/10673220802277938

[bibr60-00048674261440324] Vaughan B Goldstein MH Alikakos M , et al. (2014) Frequency of reporting of adverse events in randomised controlled trials of psychotherapy vs. psychopharmacotherapy. Comprehensive Psychiatry 55: 849–855. 24630200 10.1016/j.comppsych.2014.01.001 PMC4346151

[bibr61-00048674261440324] Vybíral Z Ogles BM Řiháček T , et al. (2024) Negative experiences in psychotherapy from clients’ perspective: A qualitative meta-analysis. Psychotherapy Research 34: 279–292. 37410872 10.1080/10503307.2023.2226813

[bibr62-00048674261440324] Wang Philip S et al. (2007). Use of mental health services for anxiety, mood, and substance disorders in 17 countries in the WHO world mental health surveys. The Lancet 370: 841–850. 10.1016/S0140-6736(07)61414-7 PMC2847360 17826169

[bibr63-00048674261440324] Wiborg JF Ben-Sliman E Michalek S , et al. (2016) Does migration affect the outcome of inpatient psychotherapy? Results from a retrospective cohort study. Journal of Psychosomatic Research 87: 81–84. 27411755 10.1016/j.jpsychores.2016.06.008

[bibr64-00048674261440324] Wong VW-H Ho FY-Y Shi N-K , et al. (2021) Lifestyle medicine for depression: A meta-analysis of randomised controlled trials. Journal of Affective Disorders 284: 203–216. 33609955 10.1016/j.jad.2021.02.012

[bibr65-00048674261440324] World Health Organisation (2022) COVID-19 pandemic triggers 25% increase in prevalence of anxiety and depression worldwide. Available at: www.who.int/news/item/02-03-2022-covid-19-pandemic-triggers-25-increase-in-prevalence-of-anxiety-and-depression-worldwide (accessed on September 25, 2025). PMC9998057 35414629

[bibr66-00048674261440324] Yacaman-Mendez D Hallgren M Forsell Y (2019) Childhood adversities, negative life events and outcomes of non-pharmacological treatments for depression in primary care: A secondary analysis of a randomised controlled trial. Journal of Psychiatric Research 110: 152–158. 30641348 10.1016/j.jpsychires.2019.01.004

[bibr67-00048674261440324] You XR Gong XR Guo MR , et al. (2024) Cognitive behavioural therapy to improve social skills in children and adolescents with autism spectrum disorder: A meta-analysis of randomised controlled trials. Journal of Affective Disorders 344: 8–17. 37802322 10.1016/j.jad.2023.10.008

[bibr68-00048674261440324] Young LM Moylan S John T , et al. (2022) Evaluating telehealth lifestyle therapy versus telehealth psychotherapy for reducing depression in adults with covid-19 related distress: The curbing anxiety and depression using lifestyle medicine (CALM) randomised non-inferiority trial protocol. BMC Psychiatry 22: 1–12. 35346115 10.1186/s12888-022-03840-3 PMC8958477

[bibr69-00048674261440324] Zhong QY Gelaye B Zaslavsky AM , et al. (2015) Diagnostic validity of the generalised anxiety disorder-7 (GAD-7) among pregnant women. PLoS One 10: e0125096. 10.1371/journal.pone.0125096 PMC4411061 25915929

